# Abdominal MRI‐Derived Liver, Visceral Fat, and Pancreatic Phenotypes Improve Prediction of Incident Type 2 Diabetes

**DOI:** 10.1002/dmrr.70235

**Published:** 2026-10-01

**Authors:** Ruotong Peng, Xuyang Diao, Yiwen Dai, Jingxin Chen, Linqing Zhu, Menghan Zhu, Xinqing Yang, Yang Pan, Yuling Liu, Darui Gao, Yanyu Zhang, Mengmeng Ji, Wuxiang Xie, Fanfan Zheng

**Affiliations:** ^1^ Department of Health and Chronic Disease Management School of Nursing Chinese Academy of Medical Sciences & Peking Union Medical College Beijing China; ^2^ Clinical Research Institute, Institute of Advanced Clinical Medicine Peking University Beijing China; ^3^ Department of Endocrinology Peking University First Hospital Beijing China; ^4^ Key Laboratory of Epidemiology of Major Diseases (Peking University) Ministry of Education Beijing China

**Keywords:** abdominal MRI, opportunistic imaging, prediction model, risk stratification, type 2 diabetes

## Abstract

**Aims:**

To evaluate whether abdominal magnetic resonance imaging (MRI)‐derived metabolic phenotypes improve the opportunistic prediction of incident type 2 diabetes (T2D) beyond clinical risk models.

**Materials and Methods:**

This study included 36,833 UK Biobank participants who underwent abdominal MRI and were free of T2D at imaging baseline. The original Cambridge Diabetes Risk Score (CDRS) was evaluated, and glycated haemoglobin (HbA1c) was added to form the clinical CDRS model. MRI phenotypes were selected using a LASSO‐based selection‐frequency analysis and added to construct the clinical CDRS‐MRI model. Model performance was assessed in an internal validation set using Harrell's C‐index, continuous net reclassification improvement (NRI), and integrated discrimination improvement (IDI).

**Results:**

During a median follow‐up of 7.38 years, 570 participants developed incident T2D. The selection‐frequency analysis retained four MRI phenotypes: liver proton density fat fraction, liver volume, visceral fat volume, and pancreas volume. In the internal validation set, C‐indices were 0.765 (95% CI, 0.731–0.800) for the original CDRS model, 0.820 (95% CI, 0.786–0.849) for the clinical CDRS model, and 0.849 (95% CI, 0.818–0.877) for the clinical CDRS‐MRI model. The clinical CDRS‐MRI model improved the C‐index by 0.084 versus the original CDRS model and by 0.029 versus the clinical CDRS model, with a continuous NRI of 56.2% (95% CI, 40.6%–70.0%) and an IDI of 0.022 (95% CI, 0.010–0.037).

**Conclusions:**

Abdominal MRI‐derived phenotypes improved the prediction of incident T2D beyond established clinical risk models. These findings support secondary use of available abdominal MRI data for metabolic risk stratification, pending external validation.

**Abbreviations**
BMI: body mass index
CDRS: Cambridge Diabetes Risk Score
CI: confidence interval
HbA1c: glycated haemoglobin
ICD‐10: International Classification of Diseases, Tenth Revision
IDI: integrated discrimination improvement
LASSO: least absolute shrinkage and selection operator
MRI: magnetic resonance imaging
NRI: net reclassification improvement
PDFF: proton density fat fraction
SD: standard deviation
T2D: type 2 diabetes
UKB: UK Biobank

## 1 Introduction

Type 2 diabetes (T2D) is a major global health challenge, contributing substantially to premature mortality, disability, and economic burden worldwide [1, 2]. Its development is typically preceded by years of subclinical metabolic deterioration, during which risk stratification may help identify individuals most likely to benefit from intensified prevention and metabolic surveillance [3, 4]. Established prediction models incorporating demographic characteristics, anthropometry, lifestyle factors, family history, medication use, and glycaemic markers have shown useful performance for diabetes risk stratification [5, 6, 7]. However, these models primarily reflect clinical risk profiles and glycaemic status and may not fully capture organ‐level metabolic abnormalities underlying diabetes susceptibility [8, 9].

Abdominal metabolic dysfunction is central to the development of T2D [10, 11]. Visceral adiposity, hepatic fat accumulation, pancreatic structural changes, and skeletal muscle fat infiltration are closely related to insulin resistance, *β*‐cell dysfunction, and progression to dysglycaemia [12]. These abnormalities may precede overt diabetes and provide information beyond body mass index (BMI) or glycated haemoglobin (HbA1c) [9, 12, 13, 14]. Abdominal magnetic resonance imaging (MRI) provides a non‐invasive, quantitative approach for characterising these metabolic phenotypes, including visceral and subcutaneous adipose tissue, liver and pancreatic fat, organ volume, and muscle fat infiltration [15, 16]. Previous imaging studies have linked individual abdominal fat depots and ectopic fat markers with dysglycaemia, insulin resistance, and incident T2D [14, 17, 18]. Nevertheless, most studies have focused on etiological associations or single imaging markers, rather than evaluating whether multi‐organ MRI‐derived phenotypes can improve diabetes risk prediction beyond established clinical and glycaemic models.

From a diabetes prevention perspective, abdominal MRI may be valuable not as a population screening test, but as a source of secondary metabolic information when imaging data are already available [14, 19]. When an abdominal MRI has already been acquired for clinical or health assessment purposes, quantitative metabolic phenotypes can potentially be extracted with minimal additional burden. This approach aligns with opportunistic imaging, in which existing imaging examinations are reused to obtain additional health information beyond the original indication [19, 20]. However, whether abdominal MRI‐derived multi‐organ metabolic phenotypes can provide incremental predictive value for incident T2D beyond clinical and glycaemic predictors remains unclear.

Therefore, using the UK Biobank (UKB) abdominal MRI cohort, we developed and internally validated an opportunistic MRI‐enhanced framework to assess whether abdominal MRI‐derived metabolic phenotypes improve the prediction of incident T2D beyond conventional risk models.

## 2 Materials and Methods

### 2.1 Study Source and Design

The UKB is a large prospective cohort that recruited more than 500,000 adults aged 40–69 years across England, Scotland, and Wales between 2006 and 2010 [21, 22, 23, 24]. At baseline, participants completed standardised questionnaires, underwent physical measurements, and provided biological samples. Health outcomes were ascertained through linkage with national health records and longitudinal follow‐up. The UKB launched its Imaging Enhancement Programme in 2014 to perform multi‐organ magnetic resonance imaging in approximately 20% of the cohort participants. This study was designed as a prediction model development and internal validation study using the UKB abdominal MRI subcohort. The UKB has ethical approval from the National Health Service North West Multi‐Centre Research Ethics Committee. All participants provided written informed consent. The analysis was conducted under UKB application number 90492 and in accordance with the principles of the Declaration of Helsinki. This study was reported in accordance with the TRIPOD + AI reporting guideline [25].

### 2.2 Study Population

Eligible participants were UKB participants who underwent the abdominal MRI assessment and had available follow‐up information on incident T2D after imaging. Participants were excluded if they had prevalent T2D before the abdominal MRI assessment, HbA1c ≥ 48 mmol/mol measured before the abdominal MRI assessment, withdrew consent, had no follow‐up after imaging, or had insufficient abdominal MRI phenotype information for model construction.

### 2.3 Outcome Definition and Follow‐Up

Incident T2D was defined using the UKB first occurrence field for International Classification of Diseases, Tenth Revision (ICD‐10) code E11, derived from mapped primary care, hospital inpatient, death registry, and self‐reported medical condition records. Incident cases were those with the first E11 occurrence after the abdominal MRI assessment. Follow‐up started at each participant's abdominal MRI assessment date and continued until incident T2D, death, loss to follow‐up, or the administrative censoring date, August 31, 2025, whichever occurred first. Endpoint definitions and data fields are shown in Supporting Information S1: Table 1.

### 2.4 Abdominal MRI Phenotypes

Abdominal MRI‐derived phenotypes were obtained from the UKB imaging enhancement [15, 16, 26]. MRI was performed at UKB imaging centres using Siemens Aera 1.5 T scanners with a standardised neck‐to‐knee Dixon protocol. Liver volume, liver proton density fat fraction (PDFF), pancreas volume, pancreas PDFF, visceral fat volume, and subcutaneous adipose tissue volume were derived from the UKB abdominal organ composition resource generated by Calico Life Sciences and the University of Westminster. Muscle fat infiltration was derived from the UKB abdominal composition resource generated by AMRA Medical. These image‐analysis pipelines have been described previously [16].

Based on the study hypothesis that T2D risk may be jointly driven by abnormal fat partitioning, ectopic fat deposition, organ structural alterations, and muscle fat infiltration [10, 13], we prespecified abdominal MRI candidate phenotypes covering four biological dimensions: (1) fat partitioning, including visceral fat volume and subcutaneous adipose tissue volume; (2) ectopic fat, including liver PDFF and pancreas PDFF; (3) organ volume, including liver volume and pancreas volume; and (4) muscle composition, represented by muscle fat infiltration. Field codes, units, and sample coverage are provided in Supporting Information S1: Table 1.

### 2.5 CDRS and HbA1c

The Cambridge Diabetes Risk Score (CDRS) was originally developed in UK primary care populations using routinely available non‐biochemical risk factors and was subsequently shown to identify individuals at increased risk of incident T2D in a prospective UK cohort [27, 28]. The National Institute for Health and Care Excellence recommends using validated risk‐assessment tools to identify individuals at high risk of T2D and lists the Cambridge diabetes risk score as an example of a computer‐based risk‐assessment tool [5]. Variables included: (1) demographics: age and sex; (2) anthropometry: body mass index (BMI); (3) clinical and medication history: current use of antihypertensive or steroid medications; and (4) lifestyle and family history: smoking status and diabetes in first‐degree relatives. The original CDRS was calculated using the published coefficients from Griffin et al. and expressed as a score ranging from 0 to 1 [27]. For time‐to‐event prediction, the calculated original CDRS score was incorporated as a single predictor in a Cox proportional hazards model, hereafter referred to as the original CDRS model. HbA1c was then added to the original CDRS score to construct the clinical CDRS model, consistent with previous diabetes prediction studies incorporating glycaemic information into clinical risk scores [29, 30]. Details of variable extraction and CDRS calculation are provided in Supporting Information S1: Method 1.

## 3 Statistical Analyses

### 3.1 Derivation–Internal Validation Split and Data Pre‐Processing

Analyses were performed using R 4.3.0 and SAS 9.4. Two‐sided *p* < 0.05 was considered statistically significant. The study population was randomly partitioned into a derivation set (70%) and an internal validation set (30%), stratified by incident T2D status. All variable selection, imputation model fitting, MRI standardisation, and Cox model estimation were performed in the derivation set. Model performance was evaluated in the internal validation set. Missing values were handled using a random forest‐based imputation method implemented in the missRanger package. Imputation models were fitted in the derivation set and applied to the internal validation set (Supporting Information S1: Table 2; Supporting Information S1: Method 2). Pairwise correlation coefficients and variance inflation factors were used to assess collinearity among MRI‐derived phenotypes.

### 3.2 Variable Selection and Model Development

To identify MRI predictors that added information beyond clinical and glycaemic risk factors, we performed a repeated‐subsampling selection‐frequency analysis based on the least absolute shrinkage and selection operator (LASSO) in the derivation set. LASSO‐penalised Cox regression was fitted in 1000 random 50% subsamples drawn without replacement. The original CDRS score and HbA1c were included as unpenalised covariates, whereas the seven MRI phenotypes were entered as penalised candidate predictors. For each MRI phenotype, the selection frequency was calculated as the proportion of iterations in which its coefficient was non‐zero. MRI variables with a prespecified selection frequency ≥ 90% were retained for the primary MRI model [31, 32]. Selected MRI predictors were ranked by their selection frequencies and incremental contribution to the clinical CDRS model. A forward accumulation analysis sequentially added selected MRI phenotypes to quantify incremental gains in discrimination. A prespecified sensitivity analysis used a relaxed selection‐frequency threshold of ≥ 85%. Further details of MRI predictor selection are provided in Supporting Information S1: Method 3.

After MRI predictor selection, four Cox proportional hazard models were developed in the derivation set and evaluated in the internal validation set. The original CDRS model included the calculated original CDRS score as a single predictor. The MRI‐only model included the selected MRI phenotypes. The clinical CDRS model included the original CDRS score and HbA1c. The clinical CDRS‐MRI model further included the selected MRI phenotypes. Cox model coefficients were estimated in the derivation set and applied without refitting to the internal validation set. The primary incremental comparison was the clinical CDRS‐MRI model versus the clinical CDRS model.

### 3.3 Model Performance Evaluation

Model performance was evaluated in the derivation and internal validation sets. Discrimination was assessed using Harrell's C‐index. The 95% confidence intervals (CIs) for C‐index and ΔC‐index were estimated using 1000 paired bootstrap resamples, and *p* values for paired C‐index comparisons were calculated using compareC. Improvement in risk classification was assessed using continuous net reclassification improvement (NRI) and integrated discrimination improvement (IDI) at the prespecified 7‐year prediction horizon. These metrics were calculated from fixed derivation‐model absolute risks with inverse probability of censoring weighting for comparisons of the clinical CDRS‐MRI model with the clinical CDRS model and the original CDRS model. The 95% CIs for NRI and IDI were estimated using 1000 paired bootstrap samples. Calibration at 7 years was assessed by comparing predicted and Kaplan‐Meier observed risks across deciles of predicted risk in the internal validation set. Overall calibration was evaluated using the observed‐to‐expected risk ratio, calibration slope, and inverse probability of censoring the weighted Brier score.

### 3.4 Subgroup Analyses

Subgroup analyses were performed to examine whether the incremental value of MRI phenotypes differed across clinically relevant strata. BMI subgroups included non‐obese participants with BMI < 30 kg/m^2^, lean participants with BMI < 25 kg/m^2^, overweight non‐obese participants with BMI 25–< 30 kg/m^2^, and obese participants with BMI ≥ 30 kg/m^2^. HbA1c subgroups included participants with HbA1c < 39 mmol/mol, 39–47 mmol/mol, < 42 mmol/mol, and 42–47 mmol/mol. In these subgroup analyses, the clinical CDRS model was used as the primary reference model.

### 3.5 Sensitivity Analyses

Several sensitivity and exploratory analyses were conducted. First, model development and validation were repeated using MRI predictors selected at a relaxed selection‐frequency threshold of ≥ 85%. Second, complete‐case analyses were performed among participants with complete data for CDRS variables, HbA1c, and all seven MRI phenotypes. Third, participants with available glucose measured before the abdominal MRI assessment in the diabetic range (≥ 11.1 mmol/L) were excluded to assess the potential influence of baseline glucose‐defined undiagnosed diabetes. Fourth, the incremental performance of the selected MRI signature was compared with that of waist circumference and waist‐to‐height ratio. The proportional hazard assumption was evaluated using Schoenfeld residuals. Further details are provided in Supporting Information S1: Method 4.

## 4 Results

### 4.1 Baseline Characteristics

The primary analysis included 36,833 UKB participants with available abdominal MRI phenotypes and without T2D at imaging baseline, after excluding those with HbA1c ≥ 48 mmol/mol. During a median follow‐up of 7.38 years, 570 participants developed incident T2D, corresponding to an incidence rate of 2.02 per 1000 person‐years. Participant selection is shown in Figure 1. The cohort was randomly split into a derivation set of 25,784 participants with 399 incident events and an internal validation set of 11,049 participants with 171 incident events. Baseline characteristics were generally similar between the derivation and internal validation sets (Table 1). Overall, the mean age was 63.62 ± 7.56 years; 52.9% of participants were female; the mean BMI was 26.36 ± 4.26 kg/m^2^; and the mean HbA1c was 34.52 ± 3.54 mmol/mol. The mean visceral fat volume was 3.84 ± 2.24 L, subcutaneous adipose tissue volume was 8.42 ± 4.09 L, liver PDFF was 4.69 ± 4.70%, pancreas PDFF was 10.41 ± 8.01%, and muscle fat infiltration was 7.28 ± 1.83%.

**FIGURE 1:**
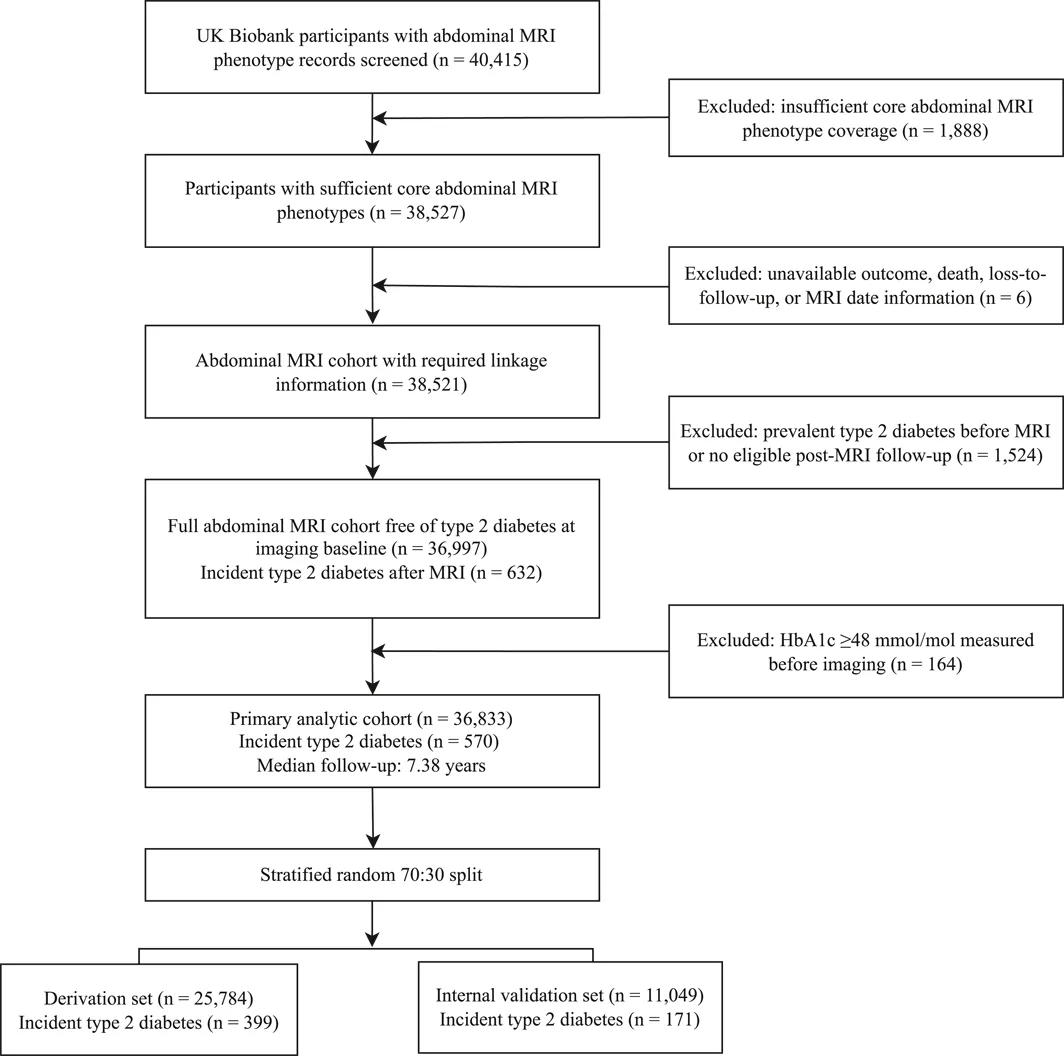
Study population flowchart. Flowchart of participant selection from the UK Biobank abdominal MRI cohort and stratified random 70:30 partitioning into derivation and internal validation sets. HbA1c, glycated haemoglobin; MRI, magnetic resonance imaging; T2D, type 2 diabetes.

**TABLE 1:** Baseline characteristics of the primary abdominal MRI cohort.

Characteristic	Derivation set	Internal validation set	*p* value
Participants	25,784	11,049	—
Incident type 2 diabetes, *n* (%)	399 (1.5)	171 (1.5)	—
Follow‐up, years	7.67 (1.65)	7.70 (1.67)	0.113
Age, years	63.62 (7.56)	63.62 (7.54)	0.992
BMI, kg/m^2^	26.36 (4.25)	26.36 (4.30)	0.969
HbA1c, mmol/mol	34.52 (3.55)	34.52 (3.52)	0.985
Sex, *n* (%)			0.595
Female	13,654 (53.0)	5817 (52.6)	
Male	12,130 (47.0)	5232 (47.4)	
Smoking status, *n* (%)			0.855
Never smoker	16,158 (62.7)	6927 (62.7)	
Former smoker	8647 (33.5)	3699 (33.5)	
Current smoker	889 (3.4)	394 (3.6)	
Missing	90 (0.3)	29 (0.3)	
Antihypertensive medication, *n* (%)			0.049
No	20,282 (78.7)	8587 (77.7)	
Yes	5493 (21.3)	2456 (22.2)	
Missing	9 (0.0)	6 (0.1)	
Family history of type 2 diabetes, *n* (%)			0.300
None	18,635 (72.3)	8066 (73.0)	
Parent or sibling	5029 (19.5)	2082 (18.8)	
Parent and sibling	763 (3.0)	334 (3.0)	
Missing	1357 (5.3)	567 (5.1)	
Steroid medication, *n* (%)			0.563
No	25,545 (99.1)	10,955 (99.1)	
Yes	237 (0.9)	94 (0.9)	
Missing	2 (0.0)	0 (0.0)	
Visceral fat volume, L	3.84 (2.24)	3.84 (2.23)	0.840
Subcutaneous adipose tissue volume, L	8.42 (4.08)	8.41 (4.12)	0.955
Muscle fat infiltration, %	7.28 (1.83)	7.28 (1.83)	0.990
Liver volume, L	1.40 (0.30)	1.40 (0.30)	0.601
Liver PDFF, %	4.70 (4.73)	4.67 (4.64)	0.570
Pancreas volume, L	0.06 (0.02)	0.06 (0.02)	0.268
Pancreas PDFF, %	10.43 (8.05)	10.36 (7.91)	0.490

*Note:* Continuous variables are presented as mean (SD), and categorical variables as *n* (%). Percentages for categorical variables were calculated using the total number of participants in each set as the denominator, with missing values shown separately. *p* values are descriptive and were calculated using observed non‐missing values.

Abbreviations: BMI, body mass index; HbA1c, glycated haemoglobin; PDFF, proton density fat fraction; SD, standard deviation.

### 4.2 Abdominal MRI Predictor Selection

Among the seven prespecified MRI‐derived phenotypes, liver PDFF, liver volume, visceral fat volume, and pancreas volume met the prespecified selection‐frequency threshold of ≥ 90%, with selection frequencies of 100.0%, 100.0%, 97.4%, and 95.3%, respectively. Muscle fat infiltration, pancreatic PDFF, and subcutaneous adipose tissue volume were selected in 86.0%, 78.6%, and 43.8% of iterations, respectively. The four retained predictors were entered in the order of liver PDFF, liver volume, visceral fat volume, and pancreas volume. Their sequential addition increased the internal validation C‐index of the clinical CDRS model from 0.820 to 0.832, 0.842, 0.846, and 0.849, respectively. Selection frequencies and forward accumulation results are shown in Figure 2. Detailed selection‐frequency results, collinearity diagnostics, and forward accumulation estimates are provided in Supporting Information S1: Tables 3–4.

**FIGURE 2:**
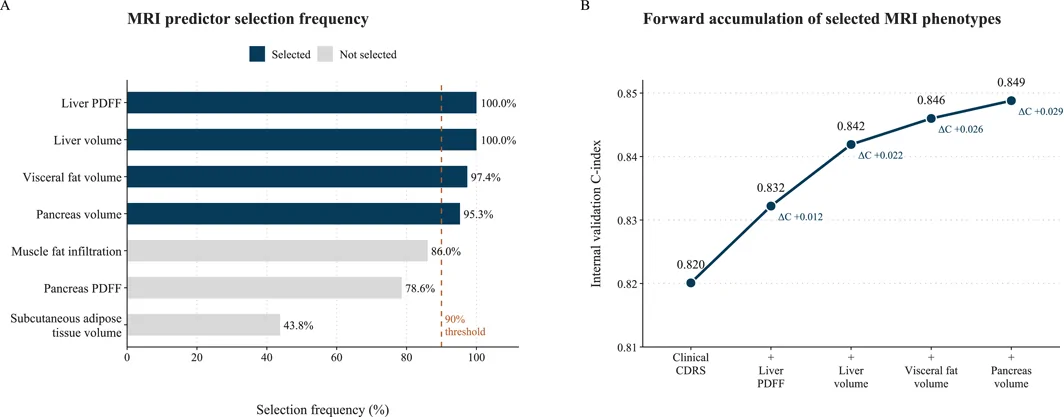
MRI predictor selection and forward accumulation. (A) Selection frequencies of seven abdominal MRI‐derived phenotypes in the LASSO‐based selection‐frequency analysis using 1000 random 50% subsamples of the derivation set. The original CDRS score and HbA1c were included as unpenalised covariates. The dashed line indicates the prespecified 90% selection‐frequency threshold. (B) Forward accumulation of the four selected MRI phenotypes beyond the clinical CDRS model in the internal validation set. ΔC values indicate cumulative improvement in C‐index relative to the clinical CDRS model. CDRS, Cambridge Diabetes Risk Score; HbA1c, glycated haemoglobin; LASSO, least absolute shrinkage and selection operator; MRI, magnetic resonance imaging; PDFF, proton density fat fraction.

### 4.3 Model Discrimination

In the internal validation set, the C‐index was 0.765 (95% CI, 0.731–0.800) for the original CDRS model, 0.792 (95% CI, 0.758–0.825) for the MRI‐only model, and 0.820 (95% CI, 0.786–0.849) for the clinical CDRS model. Adding the selected MRI phenotypes increased the C‐index to 0.849 (95% CI, 0.818–0.877). Compared with the original CDRS model, the clinical CDRS‐MRI model improved the C‐index by 0.084 (95% CI, 0.053–0.117; *p <* 0.001). When MRI phenotypes were added to the clinical CDRS model, the C‐index improved by 0.029 (95% CI, 0.018–0.041; *p <* 0.001). Detailed model performance results are presented in Table 2. Hazard ratios for predictors in the clinical CDRS‐MRI model are shown in Supporting Information S1: Table 5.

**TABLE 2:** Predictive performance of diabetes risk models in the derivation and internal validation sets.

Metrics	Derivation set	Internal validation set
Total sample size/cases	25,784/399	11,049/171
C‐index, original CDRS model	0.792 (0.772–0.811)	0.765 (0.731–0.800)
C‐index, MRI‐only model	0.800 (0.778–0.821)	0.792 (0.758–0.825)
C‐index, clinical CDRS model	0.843 (0.823–0.863)	0.820 (0.786–0.849)
C‐index, clinical CDRS‐MRI model	0.871 (0.853–0.888)	0.849 (0.818–0.877)
ΔC‐index, clinical CDRS model versus original CDRS model	0.051 (0.032–0.068)	0.055 (0.022–0.088)
*p* value for ΔC‐index comparison	< 0.001	< 0.001
ΔC‐index, clinical CDRS‐MRI model versus original CDRS model	0.079 (0.061–0.097)	0.084 (0.053–0.117)
*p* value for ΔC‐index comparison	< 0.001	< 0.001
Continuous NRI, clinical CDRS‐MRI model versus original CDRS model	84.4% (74.9%–94.7%)	78.6% (62.6%–93.3%)
IDI, clinical CDRS‐MRI model versus original CDRS model	0.078 (0.063–0.093)	0.053 (0.037–0.070)
ΔC‐index, clinical CDRS‐MRI model versus clinical CDRS model	0.028 (0.019–0.038)	0.029 (0.018–0.041)
*p* value for ΔC‐index comparison	< 0.001	< 0.001
Continuous NRI, clinical CDRS‐MRI model versus clinical CDRS model	43.9% (34.7%–53.2%)	56.2% (40.6%–70.0%)
IDI, clinical CDRS‐MRI model versus clinical CDRS model	0.028 (0.018–0.039)	0.022 (0.010–0.037)

*Note:* C‐index and ΔC‐index values are presented with 95% confidence intervals (CIs) in parentheses, estimated using 1000 paired bootstrap resamples. *p* values for paired C‐index comparisons were calculated using compareC. Continuous net reclassification improvement (NRI) and integrated discrimination improvement (IDI) were evaluated at the prespecified 7‐year prediction horizon using fixed derivation‐model absolute risks with inverse probability of censoring weighting and are presented with 95% CIs estimated using 1000 paired bootstrap resamples. The original Cambridge Diabetes Risk Score (CDRS) model included the calculated original CDRS score as a single predictor. The clinical CDRS model included the original CDRS score and glycated haemoglobin (HbA1c). The clinical CDRS‐MRI model further included four selected abdominal magnetic resonance imaging (MRI)‐derived phenotypes: liver proton density fat fraction (PDFF), liver volume, visceral fat volume, and pancreas volume.

### 4.4 Reclassification and Calibration

The clinical CDRS‐MRI model improved reclassification compared with the clinical CDRS model, with a continuous NRI of 56.2% (95% CI, 40.6%–70.0%) and an IDI of 0.022 (95% CI, 0.010–0.037) in the internal validation set. Compared with the original CDRS model, the corresponding continuous NRI was 78.6% (95% CI, 62.6%–93.3%) and the IDI was 0.053 (95% CI, 0.037–0.070). At 7 years, the Kaplan‐Meier observed risk was 1.52%, compared with mean predicted risks of 1.49% for the clinical CDRS model and 1.48% for the clinical CDRS‐MRI model. The corresponding calibration slopes were 0.936 (95% CI, 0.824–1.047) and 0.946 (95% CI, 0.848–1.044), with Brier scores of 0.0142 and 0.0140, respectively. Calibration plots showed good agreement between predicted and observed risks. Detailed reclassification results are shown in Supporting Information S1: Table 6, and calibration results are provided in Supporting Information S1: Table 7 and Supporting Information S1: Figure 1.

### 4.5 Subgroup Analyses

The incremental value of MRI was directionally consistent across the BMI subgroups. In the internal validation set, the C‐index improvement of the clinical CDRS‐MRI model over the clinical CDRS model was 0.024 among participants with BMI < 30 kg/m^2^, 0.043 among those with BMI < 25 kg/m^2^, 0.028 among those with BMI 25–< 30 kg/m^2^, and 0.064 among those with BMI ≥ 30 kg/m^2^ (Supporting Information S1: Table 8). Corresponding improvements across HbA1c‐defined subgroups were 0.045 for HbA1c < 39 mmol/mol, 0.028 for HbA1c 39–47 mmol/mol, 0.034 for HbA1c < 42 mmol/mol, and 0.058 for HbA1c 42–47 mmol/mol (Supporting Information S1: Table 9).

### 4.6 Sensitivity Analyses

The main findings were consistent across the sensitivity analyses. In complete‐case analyses, the internal validation C‐index increased from 0.830 for the clinical CDRS model to 0.855 for the clinical CDRS‐MRI model, corresponding to a ΔC‐index of 0.025 (Supporting Information S1: Table 10). The ≥ 85% selection‐frequency threshold additionally retained muscle fat infiltration, while the internal validation C‐index improvement remained 0.029 (Supporting Information S1: Table 11). Results were similar after excluding participants with glucose ≥ 11.1 mmol/L. The internal validation C‐index was 0.824 for the clinical CDRS model and 0.852 for the clinical CDRS‐MRI model, corresponding to a ΔC‐index of 0.029 (Supporting Information S1: Table 12). In the anthropometric comparison, adding waist circumference or waist‐to‐height ratio to the clinical CDRS model improved the internal validation C‐index by 0.011 and 0.010, respectively, compared with an improvement of 0.029 after adding the selected MRI signature (Supporting Information S1: Table 13). Global Schoenfeld residual tests were nonsignificant for all four models, although liver volume showed a term‐specific deviation in the MRI‐only model (*p* = 0.042) and clinical CDRS‐MRI model (*p* = 0.027; Supporting Information S1: Table 14).

## 5 Discussion

Using the UKB abdominal MRI cohort, this study developed and internally validated an MRI‐enhanced prediction framework with potential for opportunistic metabolic risk stratification. In the internal validation set, the clinical CDRS‐MRI model achieved a C‐index of 0.849, improving discrimination by 0.084 over the original CDRS model and by 0.029 over the clinical CDRS model including HbA1c. It also improved reclassification, with a continuous NRI of 56.2% and an IDI of 0.022. These findings indicate that abdominal MRI‐derived organ phenotypes capture residual metabolic risk not fully reflected by conventional clinical predictors or glycaemic status. External validation is needed before clinical implementation.

The selected MRI signature included liver PDFF, liver volume, visceral fat volume, and pancreas volume, indicating that the added predictive information reflected multi‐organ metabolic vulnerability rather than a single fat depot. Liver PDFF was retained, consistent with evidence linking hepatic fat accumulation to insulin resistance, dysglycaemia, and diabetes risk [12, 13, 17]. A study by Deng et al. reported a dose‐response association between liver MRI‐derived PDFF and oral glucose tolerance test‐defined prediabetes or T2D among patients with obesity [33]. Our study complements and extends these findings by prospectively evaluating incident T2D and the incremental predictive value of multiple abdominal MRI phenotypes beyond clinical and glycaemic predictors in a large population‐based cohort. Visceral fat volume was also retained, reflecting the established metabolic risk associated with visceral adiposity beyond BMI [13, 17]. Liver volume may reflect structural changes related to steatosis, inflammation, and metabolic stress [14]. The inverse association between pancreas volume and incident T2D is consistent with imaging and genetic evidence suggesting that a smaller pancreas size may reflect pancreatic remodelling, reduced parenchymal mass, or lower *β*‐cell functional reserve. However, MRI‐derived pancreatic volume cannot directly measure *β*‐cell mass [14, 34, 35].

In contrast, pancreatic PDFF, muscle fat infiltration, and subcutaneous adipose tissue volume were not retained in the primary MRI signature. This should not be interpreted as evidence against their biological relevance. Pancreatic PDFF may contribute to diabetes through pancreatic fat accumulation, local lipotoxicity, and impaired insulin secretion, but may add little predictive information once HbA1c, visceral adiposity, liver phenotypes, and pancreas volume are considered together [14, 18, 36]. In this penalised prediction framework, retained variables represent stable, non‐redundant predictive information rather than a ranking of biological importance.

The observed improvement in discrimination is comparable to that reported in recent biomarker‐enhanced diabetes prediction studies [29, 30]. For example, a UKB proteomics study reported a C‐index improvement of 0.022 and a continuous NRI of 42.0% after adding a selected proteomic panel to a clinical CDRS model including HbA1c [30]. Four abdominal MRI‐derived phenotypes improved the internal validation C‐index by 0.029 beyond the clinical CDRS model in our study, indicating that organ‐level imaging phenotypes can provide incremental information beyond established clinical and glycaemic predictors. Although imaging and omics approaches differ substantially in acquisition, cost, and implementation, both illustrate the potential value of adding biological information beyond routine clinical risk factors.

The subgroup findings indicate that MRI captures risk dimensions not fully reflected by BMI or HbA1c. BMI cannot distinguish visceral from subcutaneous adiposity or directly quantify ectopic fat, whereas HbA1c reflects glycaemic status rather than upstream organ‐level metabolic abnormalities [13, 14, 17]. Incremental values were observed across prespecified BMI and HbA1c subgroups. The larger improvements among participants with higher BMI or HbA1c may reflect higher event rates and greater variation in visceral adiposity, hepatic steatosis, and organ remodelling. Incremental values in lower BMI and lower HbA1c subgroups also suggest that MRI may identify adverse metabolic phenotypes before overt obesity or dysglycaemia [8, 9, 14]. These analyses were exploratory, however, and some subgroups contained relatively few events, resulting in greater uncertainty around incremental performance estimates.

These findings have practical implications for future metabolic risk stratification. Abdominal MRI is unlikely to be justified as a stand‐alone population screening tool for diabetes. The comparison with waist circumference and waist‐to‐height ratio helps clarify the role of MRI‐derived phenotypes. Simple anthropometric measures remain inexpensive and scalable for population‐level risk assessment, whereas MRI‐derived phenotypes may provide complementary information when abdominal MRI is already available. Their value therefore lies primarily in the secondary use of imaging acquired for clinical, research, or health‐assessment purposes. Phenotypes reflecting liver fat, visceral adiposity, and pancreatic structure may provide additional metabolic information beyond BMI, HbA1c, and simple measures of central adiposity. Advances in accelerated MRI acquisition, automated segmentation, and deep‐learning reconstruction may reduce scan time and the marginal cost of extracting quantitative metabolic phenotypes [37, 38]. As these technologies mature, MRI‐derived metabolic phenotyping may become increasingly feasible in selected preventive or precision risk‐stratification settings [38, 39].

## 6 Limitations

Several limitations should be acknowledged. First, although the model was evaluated in an internal validation set, both the derivation and internal validation samples were drawn from the UKB. External validation in independent cohorts with comparable abdominal MRI phenotyping, pre‐imaging clinical and glycaemic data, and long‐term ascertainment of incident T2D is therefore needed before clinical implementation. Similar limitations have been reported in recent biomarker‐ and imaging‐enhanced prediction studies, where further multicentre or external validation remained necessary [40, 41]. Second, UKB participants are generally healthier than the general population and are predominantly of European ancestry, which may limit generalisability and affect absolute risk calibration in other populations. Third, incident T2D was defined using the UKB first occurrence field for ICD‐10 code E11, rather than by systematic repeat glycaemic testing after abdominal MRI. Although participants with prevalent T2D or measured HbA1c ≥ 48 mmol/mol were excluded to reduce baseline misclassification, HbA1c alone may not identify all individuals with glucose‐defined undiagnosed diabetes, and residual misclassification remains possible. Fourth, abdominal MRI phenotypes were measured at a single imaging baseline. Because organ volumes and fat‐related phenotypes may change over time with ageing, weight change, lifestyle, medication use, and evolving metabolic status, a single measurement may introduce regression dilution and attenuate associations with incident T2D. Our findings should therefore be interpreted as evidence for risk prediction rather than causal effects of organ structure on diabetes development. Finally, missing predictor data were handled using random forest imputation, and complete‐case analyses yielded similar findings; however, bias from non‐random missingness remains possible.

## 7 Conclusions

In the UKB abdominal MRI cohort, a four‐variable MRI signature comprising liver PDFF, liver volume, visceral fat volume, and pancreas volume improved the prediction of incident T2D beyond established clinical risk models. In the internal validation set, the clinical CDRS‐MRI model increased the C‐index by 0.084 compared with the original CDRS model and by 0.029 compared with the clinical CDRS model including HbA1c, with improved continuous reclassification. These findings support the secondary use of available abdominal MRI data to extract multi‐organ metabolic information for opportunistic diabetes risk stratification. However, the model should not be interpreted as ready for population screening or routine clinical implementation. External validation and clinical utility assessment are needed to determine whether and how MRI‐derived metabolic phenotypes can be incorporated into future precision prevention strategies.

## Author Contributions

R.P., F.Z., and W.X. conceived and designed the study. R.P. and X.D. performed the statistical analysis. R.P., X.D., Y.D., J.C., L.Z., M.Z., X.Y., Y.P., Y.L., D.G., Y.Z., and M.J. were involved in data acquisition and interpretation. R.P. draughted the manuscript. F.Z. and W.X. critically revised the manuscript for important intellectual content. All authors reviewed and approved the final version of the manuscript and agree to be accountable for all aspects of the work.

## Funding

This study was supported by grants from the National Natural Science Foundation of China (82373665) and the Non‐profit Central Research Institute Fund of Chinese Academy of Medical Sciences (2021‐RC330‐001).

## Conflicts of Interest

The authors declare no conflicts of interest.

## Supporting information


Supporting Information S1

## Data Availability

The data that support the findings of this study are available from the UK Biobank database, subject to registration and application process. Further details can be found at https://www.ukbiobank.ac.uk.
