# Supplementary material for: Abdominal MRI‐Derived Liver, Visceral Fat, and Pancreatic Phenotypes Improve Prediction of Incident Type 2 Diabetes
Source: Diabetes Metab Res Rev. 2026 Oct 1;42(7):e70235. doi: 10.1002/dmrr.70235 (PMC13631372; doi:10.1002/dmrr.70235)
Supplement: Supplementary file 1 [file DMRR-42-e70235-s001.docx]

**Supplementary Material**

**Abdominal MRI-derived liver, visceral fat, and pancreatic phenotypes improve prediction of incident type 2 diabetes**

Supplementary Method 1. Variable extraction and CDRS calculation

Supplementary Method 2. Data partitioning, imputation, and standardisation

Supplementary Method 3. MRI predictor selection

Supplementary Method 4. Subgroup and sensitivity analyses

Supplementary Table 1. Variables and corresponding UK Biobank field IDs

Supplementary Table 2. Missing data before imputation in the primary cohort

Supplementary Table 3. LASSO-based selection-frequency analysis and collinearity diagnostics

Supplementary Table 4. Forward accumulation of selected MRI phenotypes beyond the clinical CDRS model

Supplementary Table 5. Hazard ratios for predictors in the clinical CDRS-MRI model

Supplementary Table 6. Reclassification in the internal validation set

Supplementary Table 7. Calibration at 7 years in the internal validation set

Supplementary Table 8. BMI subgroup discrimination in the internal validation set

Supplementary Table 9. HbA1c subgroup discrimination in the internal validation set

Supplementary Table 10. Complete-case sensitivity analysis

Supplementary Table 11. Sensitivity analysis using a ≥85% selection-frequency threshold

Supplementary Table 12. Sensitivity analysis excluding diabetic-range glucose

Supplementary Table 13. Comparison with waist circumference and waist-to-height ratio

Supplementary Table 14. Proportional hazards assumption tests

Supplementary Figure 1. Calibration at 7 years in the internal validation set

**Supplementary Method 1. Variable extraction and CDRS calculation**

For CDRS-related variables with repeated assessment instances, age, smoking status, BMI, antihypertensive medication use, family history of type 2 diabetes, and steroid medication use were preferentially obtained from imaging assessment instance 2; when unavailable, instance 1 and then instance 0 were used. Waist circumference and standing height were obtained from the same assessment instance, preferentially instance 2, followed by instance 1 and instance 0, for calculation of waist-to-height ratio. HbA1c and glucose were obtained from instance 1 when available and otherwise from instance 0. Sex was obtained from UK Biobank Field 31. The original CDRS was calculated using the published coefficients from Griffin et al. and transformed to the original 0–1 probability scale. This calculated probability was then entered as a single predictor in the Cox proportional hazards models.

**Supplementary Method 2. Data partitioning, imputation, and standardisation**

The analytic cohort was randomly partitioned into derivation and internal validation sets in a 70:30 ratio, stratified by incident T2D status. Fixed random seeds were used for reproducibility. Missing clinical variables, HbA1c, and MRI phenotypes were handled using random forest-based imputation implemented with the missRanger package. The imputation model was fitted in the derivation set and subsequently applied to the internal validation set, using predictive mean matching with five donors, 100 trees, and a maximum of three iterations. MRI-derived phenotypes were standardised using means and standard deviations estimated in the derivation set, and the same scaling parameters were applied to the internal validation set.

**Supplementary Method 3. MRI predictor selection**

MRI predictor selection was performed in the derivation set using LASSO-penalised Cox regression across 1,000 random 50% subsamples drawn without replacement. The original CDRS score and HbA1c were included as unpenalised covariates, whereas the seven MRI-derived phenotypes were penalised candidate predictors. Ten-fold cross-validation was used within each subsample, and the minimum cross-validated penalty parameter (lambda.min) was used. Because MRI variables had already been standardised using derivation-set parameters, additional standardisation within glmnet was not performed. Selection frequency was defined as the proportion of successful iterations in which the corresponding MRI coefficient was non-zero. Variables selected in ≥90% of iterations were retained for the primary model, with ≥85% used in the prespecified sensitivity analysis.

**Supplementary Method 4. Subgroup and sensitivity analyses**

BMI- and HbA1c-defined subgroup membership was determined using originally observed values rather than imputed values. Complete-case analyses retained the original derivation and validation assignments. The sensitivity analysis excluding participants with pre-imaging glucose ≥11.1 mmol/L likewise preserved the original derivation and validation split, and the MRI signature selected in the primary analysis was retained. In the anthropometric comparison, waist circumference and standing height were imputed in a separate anthropometric imputation step using clinical variables and HbA1c, without MRI phenotypes as predictors. Waist-to-height ratio was subsequently calculated as waist circumference divided by standing height, and the primary clinical CDRS and clinical CDRS-MRI models were reused without refitting.

**Supplementary Table 1. Variables and corresponding UK Biobank field IDs**

| **Variable** | **Field ID(s)** | **Description** |
| --- | --- | --- |
| Incident type 2 diabetes | 130708 | Date of first reported ICD-10 E11 |
| Death | 40000 | Date of death |
| Loss to follow-up | 191 | Date believed lost to follow-up |
| Abdominal MRI date | 53 | Imaging assessment date |
| Visceral fat volume | 21085 | Abdominal MRI phenotype |
| Subcutaneous adipose tissue volume | 21086 | Abdominal MRI phenotype |
| Muscle fat infiltration | 22435 | Abdominal MRI phenotype |
| Liver volume | 21080 | Abdominal MRI phenotype |
| Liver PDFF | 21088 | Abdominal MRI phenotype |
| Pancreas volume | 21087 | Abdominal MRI phenotype |
| Pancreas PDFF | 21090 | Abdominal MRI phenotype |
| Age | 21003 | Age at assessment |
| Sex | 31 | Sex |
| BMI | 21001 | Body mass index |
| Smoking | 20116 | Smoking status |
| HbA1c | 30750 | Glycated haemoglobin, mmol/mol |
| Glucose | 30740 | Glucose, mmol/L |
| Waist circumference | 48 | Waist circumference, cm |
| Standing height | 50 | Standing height, cm |
| Waist-to-height ratio | Derived from Fields 48 and 50 | Calculated as waist circumference divided by standing height |
| Antihypertensive medication | 6177, 6153 | Medication indicator |
| Family history of type 2 diabetes | 20107, 20110, 20111 | First-degree family history |
| Steroid medication | 20003 | Regular medication codes |

**Note:** ICD-10, International Classification of Diseases, Tenth Revision; BMI, body mass index; HbA1c, glycated haemoglobin; MRI, magnetic resonance imaging; PDFF, proton density fat fraction.

**Supplementary Table 2. Missing data before imputation in the primary cohort**

| **Variable** | **Available N** | **Missing N** | **Missing proportion** |
| --- | --- | --- | --- |
| Age | 36,833 | 0 | 0.00% |
| Sex | 36,833 | 0 | 0.00% |
| BMI | 36,833 | 0 | 0.00% |
| Smoking | 36,714 | 119 | 0.32% |
| Antihypertensive medication | 36,818 | 15 | 0.04% |
| Family history of type 2 diabetes | 34,909 | 1,924 | 5.22% |
| Steroid medication | 36,831 | 2 | 0.01% |
| HbA1c | 34,672 | 2,161 | 5.87% |
| Visceral fat volume | 36,833 | 0 | 0.00% |
| Subcutaneous adipose tissue volume | 36,833 | 0 | 0.00% |
| Muscle fat infiltration | 36,190 | 643 | 1.75% |
| Liver volume | 36,833 | 0 | 0.00% |
| Liver PDFF | 28,883 | 7,950 | 21.58% |
| Pancreas volume | 35,514 | 1,319 | 3.58% |
| Pancreas PDFF | 28,081 | 8,752 | 23.76% |

**Note:** Missingness is shown for the primary cohort before imputation. Imputation models were fitted in the derivation set and applied to the internal validation set. BMI, body mass index; HbA1c, glycated haemoglobin; PDFF, proton density fat fraction.

**Supplementary Table 3. LASSO-based selection-frequency analysis and collinearity diagnostics**

| **MRI phenotype** | **Selection frequency** | **VIF** | **Selected at ≥90%** |
| --- | --- | --- | --- |
| Liver PDFF | 100.0% | 1.713 | Yes |
| Liver volume | 100.0% | 1.876 | Yes |
| Visceral fat volume | 97.4% | 2.356 | Yes |
| Pancreas volume | 95.3% | 1.206 | Yes |
| Muscle fat infiltration | 86.0% | 1.520 | No |
| Pancreas PDFF | 78.6% | 1.670 | No |
| Subcutaneous adipose tissue volume | 43.8% | 1.647 | No |

**Note:** Selection frequency was calculated across 1,000 random 50% derivation-set subsamples. The original CDRS score and HbA1c were included as unpenalised covariates, whereas MRI phenotypes were penalised candidate predictors. LASSO, least absolute shrinkage and selection operator; VIF, variance inflation factor; CDRS, Cambridge Diabetes Risk Score; HbA1c, glycated haemoglobin; PDFF, proton density fat fraction. The maximum pairwise Pearson |r| among MRI phenotypes was 0.585.

**Supplementary Table 4. Forward accumulation of selected MRI phenotypes beyond the clinical CDRS model**

| **Step** | **Added MRI phenotype** | **MRI phenotypes in model** | **Internal validation C-index** | **Stepwise ΔC-index** | **Derivation LRT P value** |
| --- | --- | --- | --- | --- | --- |
| 0 | None | None | 0.820 | — | — |
| 1 | Liver PDFF | Liver PDFF | 0.832 | 0.012 | <.001 |
| 2 | Liver volume | Liver PDFF + liver volume | 0.842 | 0.010 | <.001 |
| 3 | Visceral fat volume | Liver PDFF + liver volume + visceral fat volume | 0.846 | 0.004 | <.001 |
| 4 | Pancreas volume | Liver PDFF + liver volume + visceral fat volume + pancreas volume | 0.849 | 0.003 | <.001 |

**Note:** The clinical CDRS model was the base model at step 0. Stepwise ΔC-index values are relative to the immediately preceding model. LRTs were performed in the derivation set. CDRS, Cambridge Diabetes Risk Score; LRT, likelihood ratio test; PDFF, proton density fat fraction. Stepwise ΔC-index values were calculated from unrounded C-index estimates.

**Supplementary Table 5. Hazard ratios for predictors in the clinical CDRS-MRI model**

| **Predictor** | **Hazard ratio (95% CI)** | **P value** |
| --- | --- | --- |
| Original CDRS score | 5.483 (3.451–8.711) | <.001 |
| HbA1c, per mmol/mol | 1.278 (1.245–1.312) | <.001 |
| Liver PDFF, per 1 SD | 1.181 (1.093–1.275) | <.001 |
| Liver volume, per 1 SD | 1.230 (1.122–1.348) | <.001 |
| Visceral fat volume, per 1 SD | 1.276 (1.137–1.432) | <.001 |
| Pancreas volume, per 1 SD | 0.851 (0.783–0.925) | <.001 |

**Note:** The original CDRS score was entered on its native 0–1 scale; HbA1c is reported per 1 mmol/mol; MRI phenotypes are reported per 1-SD increase after standardisation using derivation-set means and standard deviations. CDRS, Cambridge Diabetes Risk Score; CI, confidence interval; HbA1c, glycated haemoglobin; MRI, magnetic resonance imaging; PDFF, proton density fat fraction; SD, standard deviation.

**Supplementary Table 6. Reclassification in the internal validation set**

| **Comparison** | **Continuous NRI (95% CI)** | **IDI (95% CI)** |
| --- | --- | --- |
| Clinical CDRS-MRI model vs clinical CDRS model | 56.2% (40.6%–70.0%) | 0.022 (0.010–0.037) |
| Clinical CDRS-MRI model vs original CDRS model | 78.6% (62.6%–93.3%) | 0.053 (0.037–0.070) |

**Note:** Continuous NRI and IDI were evaluated at the prespecified 7-year prediction horizon using fixed derivation-model absolute risks with inverse probability of censoring weighting. The 95% CIs were estimated using 1,000 paired bootstrap resamples. NRI, net reclassification improvement; IDI, integrated discrimination improvement; CI, confidence interval; CDRS, Cambridge Diabetes Risk Score.

**Supplementary Table 7. Calibration at 7 years in the internal validation set**

| **Model** | **N** | **Events by 7-year horizon** | **Mean predicted 7-y risk** | **KM observed 7-y risk** | **O/E ratio** | **Mean absolute calibration error** | **Calibration slope (95% CI)** | **IPCW Brier score** |
| --- | --- | --- | --- | --- | --- | --- | --- | --- |
| Original CDRS model | 11,049 | 165 | 1.52% | 1.52% | 0.999 | 0.0034 | 0.914 (0.768–1.060) | 0.0147 |
| MRI-only model | 11,049 | 165 | 1.49% | 1.52% | 1.023 | 0.0026 | 0.973 (0.858–1.088) | 0.0146 |
| Clinical CDRS model | 11,049 | 165 | 1.49% | 1.52% | 1.024 | 0.0024 | 0.936 (0.824–1.047) | 0.0142 |
| Clinical CDRS-MRI model | 11,049 | 165 | 1.48% | 1.52% | 1.024 | 0.0031 | 0.946 (0.848–1.044) | 0.0140 |

**Note:** Observed 7-year risk was estimated using the Kaplan–Meier method. The ideal O/E ratio and calibration slope are 1. The Brier score was calculated using inverse-probability-of-censoring weighting. KM, Kaplan–Meier; O/E, observed-to-expected; IPCW, inverse probability of censoring weighting; CI, confidence interval; CDRS, Cambridge Diabetes Risk Score.

**Supplementary Table 8. BMI subgroup discrimination in the internal validation set**

| **Subgroup** | **N / events** | **Clinical CDRS C-index** | **Clinical CDRS-MRI C-index** | **ΔC-index** |
| --- | --- | --- | --- | --- |
| Full abdominal MRI cohort | 11,049 / 171 | 0.820 | 0.849 | 0.029 |
| BMI <30 kg/m² | 9,161 / 98 | 0.803 | 0.827 | 0.024 |
| BMI <25 kg/m² | 4,608 / 28 | 0.766 | 0.809 | 0.043 |
| BMI 25–<30 kg/m² | 4,553 / 70 | 0.791 | 0.819 | 0.028 |
| BMI ≥30 kg/m² | 1,888 / 73 | 0.749 | 0.813 | 0.064 |

**Note:** Results are from the internal validation set. BMI subgroups were prespecified and are not all mutually exclusive; BMI <30 kg/m² includes the BMI <25 kg/m² and BMI 25–<30 kg/m² subgroups. BMI, body mass index; CDRS, Cambridge Diabetes Risk Score; MRI, magnetic resonance imaging.

**Supplementary Table 9. HbA1c subgroup discrimination in the internal validation set**

| **Subgroup** | **N / events** | **Clinical CDRS C-index** | **Clinical CDRS-MRI C-index** | **ΔC-index** |
| --- | --- | --- | --- | --- |
| Full abdominal MRI cohort | 11,049 / 171 | 0.820 | 0.849 | 0.029 |
| HbA1c <39 mmol/mol | 9,405 / 86 | 0.763 | 0.808 | 0.045 |
| HbA1c 39–47 mmol/mol | 1,005 / 73 | 0.728 | 0.756 | 0.028 |
| HbA1c <42 mmol/mol | 10,224 / 126 | 0.795 | 0.829 | 0.034 |
| HbA1c 42–47 mmol/mol | 186 / 33 | 0.614 | 0.672 | 0.058 |

**Note:** Results are from the internal validation set. HbA1c subgroups were prespecified and are not all mutually exclusive. HbA1c subgroup analyses were restricted to participants with observed pre-imaging HbA1c; therefore, subgroup counts do not sum to the full internal validation cohort. HbA1c, glycated haemoglobin; CDRS, Cambridge Diabetes Risk Score; MRI, magnetic resonance imaging.

**Supplementary Table 10. Complete-case sensitivity analysis**

| **Model** | **Internal validation C-index** | **ΔC-index vs clinical CDRS model** |
| --- | --- | --- |
| Original CDRS model | 0.758 | −0.072 |
| MRI-only model | 0.778 | −0.052 |
| Clinical CDRS model | 0.830 | Reference |
| Clinical CDRS-MRI model | 0.855 | 0.025 |

**Note:** Complete-case sample sizes were 15,873 participants with 207 events in the derivation set and 6,797 participants with 91 events in the internal validation set. CDRS, Cambridge Diabetes Risk Score; MRI, magnetic resonance imaging.

**Supplementary Table 11. Sensitivity analysis using a ≥85% selection-frequency threshold**

| **Model** | **Internal validation C-index** | **ΔC-index vs clinical CDRS model** |
| --- | --- | --- |
| Original CDRS model | 0.765 | −0.055 |
| MRI-only model | 0.794 | −0.026 |
| Clinical CDRS model | 0.820 | Reference |
| Clinical CDRS-MRI model | 0.849 | 0.029 |

**Note:** The ≥85% selection-frequency threshold retained five MRI phenotypes: liver PDFF, liver volume, visceral fat volume, pancreas volume, and muscle fat infiltration. CDRS, Cambridge Diabetes Risk Score; MRI, magnetic resonance imaging; PDFF, proton density fat fraction.

**Supplementary Table 12. Sensitivity analysis excluding diabetic-range glucose**

| **Model** | **Internal validation C-index** | **ΔC-index vs clinical CDRS model** |
| --- | --- | --- |
| Original CDRS model | 0.764 | −0.060 |
| MRI-only model | 0.793 | −0.031 |
| Clinical CDRS model | 0.824 | Reference |
| Clinical CDRS-MRI model | 0.852 | 0.029 |

**Note:** Participants with available glucose ≥11.1 mmol/L before imaging were excluded. The sensitivity cohort included 36,826 participants and 570 events; the derivation set included 25,779 participants with 399 events and the internal validation set included 11,047 participants with 171 events. CDRS, Cambridge Diabetes Risk Score; MRI, magnetic resonance imaging.

**Supplementary Table 13. Comparison with waist circumference and waist-to-height ratio**

| **Model** | **Internal validation C-index** | **ΔC-index vs clinical CDRS model** |
| --- | --- | --- |
| Clinical CDRS model | 0.820 | Reference |
| Clinical CDRS + waist circumference | 0.832 | 0.011 |
| Clinical CDRS + waist-to-height ratio | 0.830 | 0.010 |
| Clinical CDRS-MRI model | 0.849 | 0.029 |

**Note:** This was a separate anthropometric comparison. The clinical CDRS and clinical CDRS-MRI models were identical to those in the primary analysis; waist circumference and waist-to-height ratio were added separately to the clinical CDRS model. CDRS, Cambridge Diabetes Risk Score; MRI, magnetic resonance imaging.

**Supplementary Table 14. Proportional hazards assumption tests**

| **Model** | **Term** | **Chi-square** | **P value** |
| --- | --- | --- | --- |
| Original CDRS model | Original CDRS score | 0.167 | .683 |
| Original CDRS model | Global test | 0.167 | .683 |
| MRI-only model | Liver PDFF | 2.028 | .154 |
| MRI-only model | Liver volume | 4.130 | .042 |
| MRI-only model | Visceral fat volume | 0.330 | .566 |
| MRI-only model | Pancreas volume | 0.394 | .530 |
| MRI-only model | Global test | 5.619 | .229 |
| Clinical CDRS model | Original CDRS score | 0.046 | .831 |
| Clinical CDRS model | HbA1c | 0.007 | .934 |
| Clinical CDRS model | Global test | 0.064 | .968 |
| Clinical CDRS-MRI model | Original CDRS score | 0.003 | .960 |
| Clinical CDRS-MRI model | HbA1c | 0.098 | .755 |
| Clinical CDRS-MRI model | Liver PDFF | 2.389 | .122 |
| Clinical CDRS-MRI model | Liver volume | 4.910 | .027 |
| Clinical CDRS-MRI model | Visceral fat volume | 0.573 | .449 |
| Clinical CDRS-MRI model | Pancreas volume | 0.771 | .380 |
| Clinical CDRS-MRI model | Global test | 6.565 | .363 |

**Note:** Tests were based on Schoenfeld residuals. Global tests were nonsignificant for the original CDRS, MRI-only, clinical CDRS, and clinical CDRS-MRI models. Liver volume showed term-specific evidence of non-proportionality in the MRI-only model (P=.042) and clinical CDRS-MRI model (P=.027). CDRS, Cambridge Diabetes Risk Score; MRI, magnetic resonance imaging; PDFF, proton density fat fraction.

**Supplementary Figure 1. Calibration at 7 years in the internal validation set**

**Note:** Observed and predicted 7-year risks are shown across deciles of predicted risk for the original CDRS model, MRI-only model, clinical CDRS model, and clinical CDRS-MRI model. Observed risks were estimated using the Kaplan–Meier method. Calibration slope and observed-to-expected (O/E) ratio are shown for each model. CDRS, Cambridge Diabetes Risk Score; MRI, magnetic resonance imaging.
